# Phenotype Variability Mimicking as a Process for the Test and Optimization of Dynamic Facade Systems

**DOI:** 10.3390/biomimetics7030085

**Published:** 2022-06-26

**Authors:** Ana Cocho-Bermejo, Maria Vogiatzaki

**Affiliations:** Faculty of Science and Engineering, Anglia Ruskin University, Chelmsford CM1 1SQ, UK; maria.vogiatzaki@aru.ac.uk

**Keywords:** artificial neural networks, evolutionary computation, genetic algorithms, pareto frontier, intelligent facade, insulation optimization, artificial intelligence, complex systems, phenotype

## Abstract

A genetic algorithm and an artificial neural network are deployed for the design of a dynamic multi-layered façade system that adapts in real-time to different weather and occupants’ needs scenarios. The outputs are a set of different performances of the façade insulation cushions, optimized by the previous run of the genetic algorithm. A façade system of ETFE cushions is considered for them to learn from environmental data models. Each façade cushion is set up as an artificial neuron that is linked to the behavior and temperature of the others. The proposed outputs are a set of different performances of the façade system that are optimized through running the genetic algorithm. Façade neurons are configured as genes of the system that is abstractly represented on a digital model. The computational model manages cushion patterns’ performances through several phenotypical adaptations, suggesting that the proposed facade system maximizes its thermal efficiency in different scenarios.

## 1. Introduction

For contemporary architecture to be topical and relevant, it needs to be adaptive, responsive, dynamic, and agile. These assets need to render architecture faster and more alert, not only to follow the rapid pace at which changes happen, but to supersede it.

The capacity “parametricism” offers to generate variability and facilitate the management of parameters has been thought to offer a fertile ground for an architecture of adaptivity, responsiveness, and agility. Time is one of the most important parameters or variables of architecture, and performativity—or in other words, dynamic performance—as another one, are two notions that propose a new aesthetics of architecture, which is in a perpetual transformation while continuously performing in time.

The current research studies the mechanisms whereby this new aesthetic a building façade acquires when it adapts, or performs, in response to solar gains.

The evolutionary computation processes implemented in this research are developed over an abstract digital model of the facade system proposed by the authors in [1] following an earlier research project. As mentioned in the abstract, a dynamic multilayered membrane was designed as a dynamic facade system able to adapt in real-time to different weather and occupant needs scenarios. As so, the proposed multi-layered facade system will propose a new aesthetics when, in its effort to respond to different thermal scenarios, the performance of the facade ETFE components are optimized.

Therefore, its aesthetic and mechanical properties develop an affective relationship in a continuum, a complex system in unison, while providing options for sun-shading and control of solar gains.

To test this hypothesis, the article aims to study the implementation of an intelligent algorithm that will help in the decision-making process of thermal real-time optimization and, by doing so, also in the final form of the facade through the different patterns created by the insulation cushions proposed. In order to develop this idea, an integrated dynamic system has already been designed and tested physically by the authors in [1].

The research is organized as follows: state-of-the-art and literature review are developed within Section 2. Section 3 is devoted to the explanation of the methodology, and Section 4 presents the research results. Conclusions and further research are discussed in Section 5.

## 2. Conceptual Framework and Literature Review

### 2.1. Dynamic Parametrics: Emergence and Continuous Variation

Architecture must be perpetually transformative. The architect as a geometer is no longer relevant, and neither are the Euclidean geometrically defined aesthetics. The power of algorithms that generate patterns of activities is capable of accommodating, ameliorating, and rethinking architecture away from anthropocentric appreciations.

The algorithms followed by ants to construct their colonies or the spectrum of colors birds see are a few of the abilities—intelligences that humans must learn from and use effectively. The study of biology to appreciate the genotypes, or the inner mechanisms of entities, that then are manifested physically as phenotypes, is what governs the architecture of tomorrow and its emergent aesthetics.

Design processes demand the participation and coordination of information and tasks [2]. Their complexity is imperative to be dealt with only with human intelligence and sensorial domains alone. Machines’ and algorithms’ inner power to be creative while learning is the way forward.

Coding and decoding lead to variations that offer their own aesthetics away from top-down human determinism. Interested in genotype and not phenotype away from the power of form, but more in the information a form includes and becomes one of the infinite variations of the same genotype that adapts, transforms, and changes.

In the Gothic [3] aesthetics of variations, aesthetics emerges from the resistance to loads and the distribution of forces, as in Chuck Hoberman’s [4] work, where the aesthetics of variation could emerge from the building’s resistance to undesired solar gains and direct exposure to sunlight.

Evolutionary computation is what can transform a building into an adaptive machine with its own intrinsic and variable aesthetics. It will be a building machine with its innate nonhuman bias that comes from its own machinic nature that can learn to learn in its own unique way.

In the first decade of the new millennium, the architectural discourse was talking about architecture that had to be agile and adaptable. All creative disciplines are deeply fascinated by the deployment of artificial intelligence in their creative processes.

“Parametricism” [5], a term that was very popular early in the new millennium, has been questioned by scholars such as Bernard Cache. Cache considers it to be a pre-consecrated “ism”, as he states in his work on the obsolescence of the parametric reflected in “After Parametrics” already by 2009 [6].

Nowadays, consensus about the fundamentals of future architectural design processes seems to have been reached as follows: framed as continuous variation and intelligent emergence [7]. The introduction of dynamism implies a shift from a passive neutral space to an active space of interactions. Architecture can be conceptualized and characterized by forces that can be crystallized into forms, as already stated by P. Eleni et al. in their studies about interaction and multi-agent systems in [8].

At the dawn of the 21st-century architects, such as Gregg Lynn, NOX, DECOI, and Marcos Novak were already interested in developing dynamic and evolving design techniques. As Greg Lynn [9] stated as early as 1999 in his “Animated form,” animation implies the evolution of form and its shaping forces. Moreover, Kwinter [10] uses Conrad Waddington’s Epigenetic Landscape and describes it as the relationship between an evolving form and its environment—the stimuli in that environment—a topic upon which D’Arcy Thompson [11] already elaborated in 1942. In addition to that idea, in the 2000s, Manovich asserted that we are shifting from the idea of the document to the idea of performance [12].

As everything generated digitally is variable; it is due to this variability, produced with emergent form-finding methods, that the current research suggests that new aesthetics come about.

### 2.2. Time-Based Dynamism of a New Aesthetics. Phenotype Inspirational Adaptability Processes for Building Technology System Testing

Parametricism has been deployed in two ways. One way is in the design process of a complex form so that, with the use of non-Euclidean geometry, the definition of all coordinates of a surface could be translated from software to hardware for that complexity to be fabricable. This approach ceases the use of parametricism beyond the completion of a complex form, what we could call a static approach to parametricism. Another way is in the full potential of the principles of parametricism that can define the dynamic performance and data-driven criteria towards new emergent aesthetics. Burry [13] suggests that the random approach to parametricism, as an end in itself, is not compatible with the responsibility architecture and its processes bear, which rely, to a large extent, on controlling the design process. We could call the responsible approach to parametricism dynamic.

In 2003, M. Guggenheim et al. [14] chose the title *“Joy in repetition makes the future disappear”* for an article they contributed to the publication in Science and technology studies in academia, titled “Looking back, ahead” [14]. Throughout their argument about science and technology education in academia, they stated by referring to Luhmann that “we are forcing buildings to make future operational decisions based on the past decisions made during their design process”. Thus, they lay the foundations for the differentiation of an intelligent dynamism whose algorithm of control is present as early as in the commencement of architectural design processes and is therefore inherent to and inseparable from the design entity itself.

In this omnipresent ongoing debate, this research proposes to elaborate on dynamic parametricism approaches to architecture, as stated by the authors in [15]. “Time” is considered as the new parameter that would reinstate the dynamism of the design processes toward a new parametric aesthetics, as has been already stated by the authors [1] in previous publications.

Dynamic parametric design processes generate a genre of architecture in which the initial design inputs may vary throughout the building’s lifetime. It is a relational, associative logic embedded in the design process through computation that attributes to the building properties of variability in operations and aesthetics.

### 2.3. Nature Processes Mimicking in Architectural Design: Evolutionary Computation and Artificial Neurons

Bottom-up robotics and evolutionary processes nurture artificial intelligent systems that simulate emergent and generative properties of natural processes, obtaining well-adapted and efficient forms. Artificial intelligence (AI) processes based on biology as a source for intelligent performance have been proposed in architecture since the 2000s.

The use of ANNs in architectural design is nevertheless quite recent. Agent-based modeling has a long history as Space Syntax was one of the first processes to implement artificial intelligence-based algorithms in spatial analysis and design; ANN’s history is as short as 10 years.

Examples that can be found nowadays are fundamentally based on the following three approaches:

Firstly, ANN was deployed to study building organization patterns and space distribution to generate further patterns to offer alternative space organization. In SIGMAUD 2017, Phelan et al. [16] presented a method for predicting meeting-room utilization based on a 56-building usage-training model.

Secondly, ANN is a method for substituting simulation along with the initial design phases for decision-making purposes. As so, Anwar et al. [17] propose the use of artificial neural networks for structural performance and, combined with a genetic algorithm, the optimization of its cost in tall buildings.

In 2012, Behboudi et al. [18] proposed a test to automate the design process of a building using artificial neural networks to replace simulation processes. Combined with multi-objective genetic algorithms for searching the solution space, they consider design objectives of thermal comfort, structural properties, and cost.

Thirdly, ANN is an approach to studying façade behavior regarding its interaction with the changing environment [19]. A well-known example is AEDAS’ Al Bahar Towers, which were intended to include machine learning in their behavior. The towers’ façades are composed of a series of stretched PTFE. Panels that operate by a linear module, which opens and closes progressively once a day. Responding to a pre-programmed sequence that was calculated to prevent and filter direct sunlight exposure, it did not finally implement any intelligent artificial decision-making processes.

The authors of this research believe that the use of artificial neural networks can have a great impact and could help implement dynamic parametrics in the architectural workflow, especially when combined with evolutionary computation towards an adaptive, responsive, and agile architecture.

No real-time working dynamic facade systems have been successfully implemented yet in architecture. The most famous example, the AEDAS tower, previously mentioned attempt, was finally implemented as a dynamic façade but with no intelligence optimization in real-time. Finally, it was implemented just as a stimulus-reaction-based behavior for the façade sun-protecting units.

**Figure 1:**
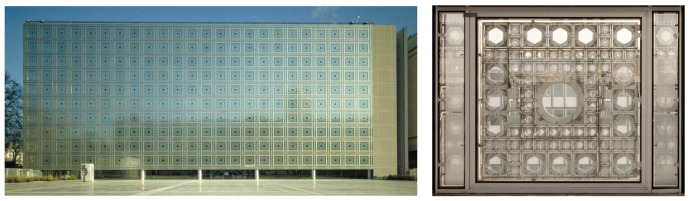
Left, Arab du Monde institute façade. Right, façade unit. Source: authors.

The first known example of the aesthetics of a building based on the dynamics of its façade is the Arab du Monde Institute, designed by Jean Nouvel in Paris in 1988 (Figure 1). The proposed façade was composed of a series of mechanical units able to react to the sunlight, closing themselves based on that stimulus being sensed.

Despite being the first modern attempt developed as old as in the 1980s, no other built example has been achieved that has more than a stimulus-reaction behavior.

**Figure 2:**
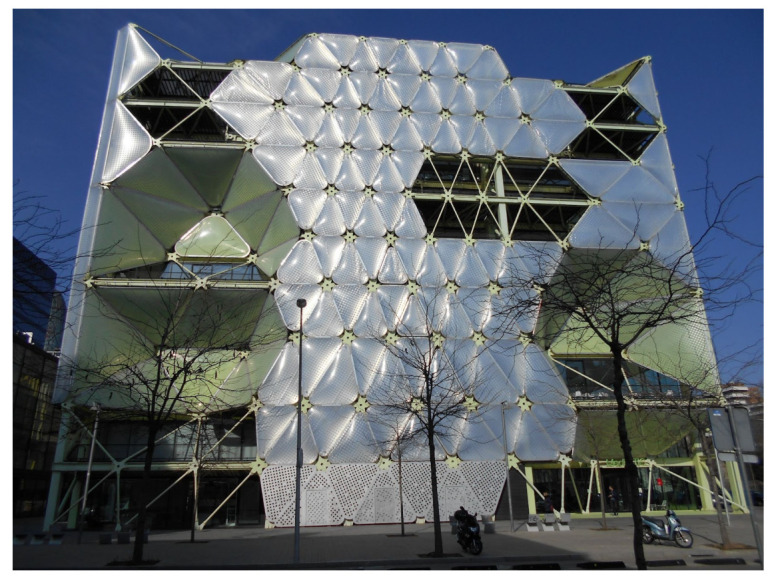
MediaTIC building ETFE cushions façade. Source: authors.

In 2010, the following well-known example was built: the mediaTIC building in Barcelona by architect E. Ruiz Geli (Figure 2). In this case, units of the façade were proposed as insulation dynamic units. Nevertheless, once again, the behavior and opening/closing decision-making process were guided by an individual stimulus-reaction process linked to a sensor and microchip installed on every cushion of the system.

To date, there are no built examples of dynamic façades on which a general behavior pattern for the units has been implemented successfully. Due to the difficulty of implementing behaviors on which every individual unit of the façade takes its decisions depending on the other units’ decisions and requirements, no general pattern-optimized facades have been built so far.

The authors’ research aims toward the resolution of that particular issue. As so, in the proposed case study, the behavior of the different units of the façade is studied as part of a whole, understanding the whole as the complex system of the 100 ETFE cushions of the façade configured. No individual stimulus-reaction behavior for the façade units is allowed.

The proposal tries to develop a better understanding of the path that must be followed through artificial intelligence for this type of façade system to work as a whole and not as the sum of one hundred individual sensing units.

The ability of a building’s envelope to be able to understand and adapt to environmental and users’ needs is the fundamental aim of a series of case studies developed by the authors trying to evaluate how general façade pattern performance can improve building mechanics and environmental efficiency.

This is precisely the type of testing the below case study aims to perform.

## 3. Case Study Facade System Layers and Components

**Figure 3:**
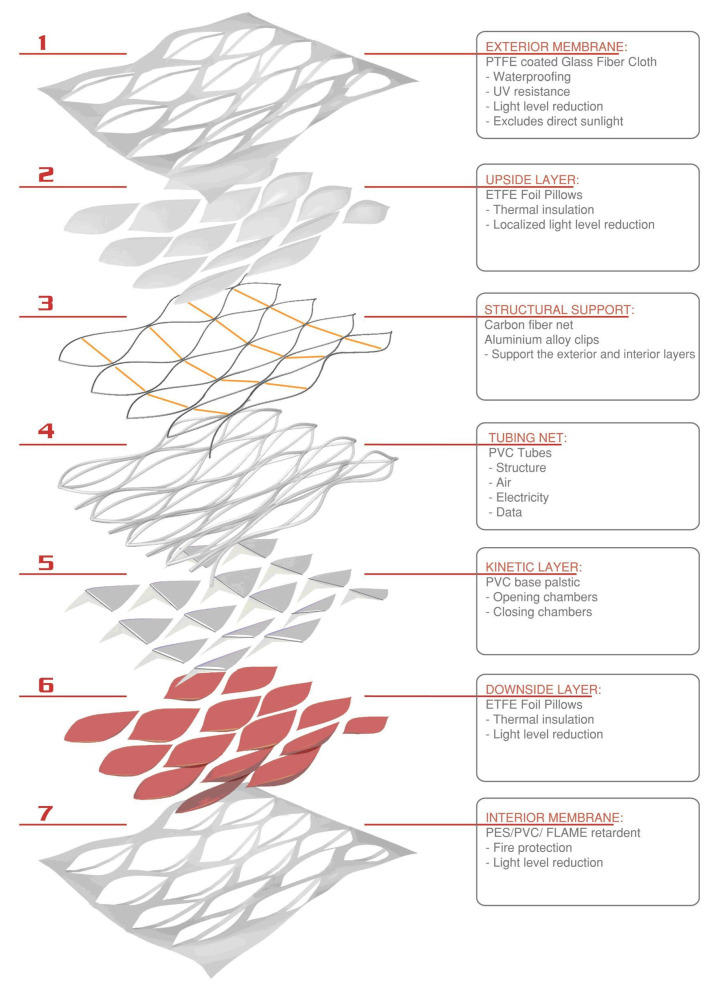
Facade system layers description.

**Figure 4:**
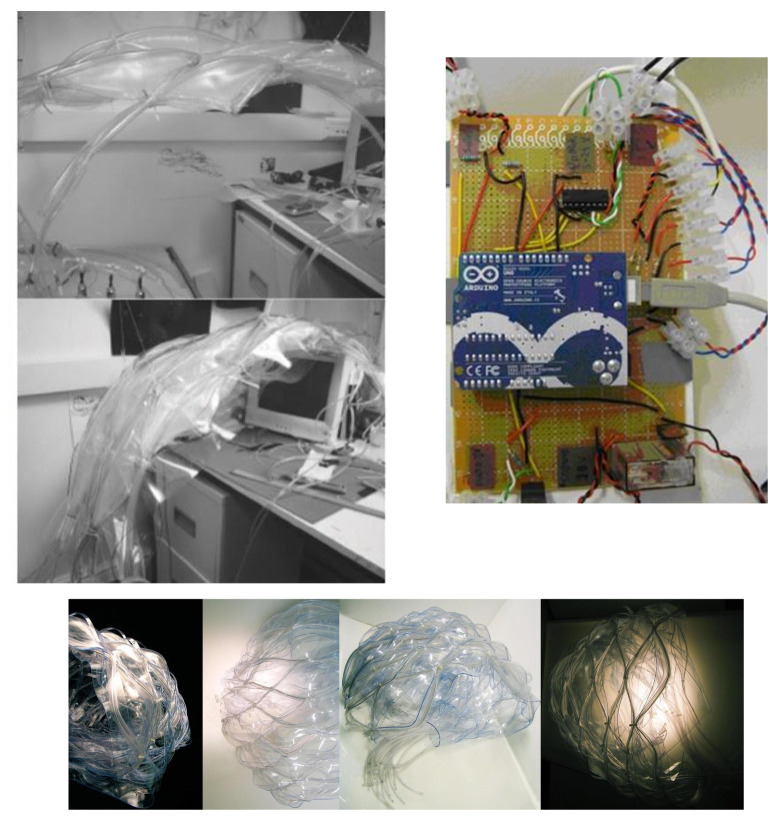
(**top left**) Dynamic membrane models at 1:5. (**top right**) Connections, Arduino for the performance of the 1:5 scale model; Circuit composed by: solenoid valve (18 V), motor (3 V), and several LEDs and several resistances. (**Below**) Static models scale 1:20.

The comprehensive design of the facade multi-layered system was organized in three phases. The two initial phases were devoted to designing and testing the façade’s physical system (Figure 3) and its real-time movement viability through a series of 1:5 scale static and dynamic Arduino controlled mock-ups (Figure 4).

The multi-layered-facade system is composed of a set of ETFE cushions able to open and close independently for different thermal requirements. The cushions’ system includes an intermediate graphically patterned layer that can be deployed for shading purposes.

In this research, the phase focusing on evolutionary computation implementation for the decision-making processes of the system along its lifetime is presented.

## 4. Methodology for Designing the Abstract Computational Model

### 4.1. General Approach and Initial Concerns

By means of an abstract computational model for the system to monitor the facade openings, the system [20] is computationally configured to have two goals at two different scales.

First, it has the following global goal: a state simulation for insulation optimization configuring the open-close cushion pattern of the entire membrane. Secondly, it has several local goals for the sun-shading behavior of the individual cushions.

The artificial intelligence approach used in the current study is the one in which, in a continuous loop, an intelligent agent receives data from the environment through sensors. It can change its state (or not) by interacting with the environment through actuators (Figure 5).

The intelligent agent in this case is the material system within a perception-action circle, benefiting from the adaptability property of the system. Artificial intelligent algorithms are studied as a method for uncertainty management with the aim of finding actions for an agent (1).

act = AgentFn(percept)(1)

Our particular agent will need to make decisions depending on external weather data in order to optimize insulation and sun-shading control. It will, therefore, need to not only externally read weather data but also internal temperatures required by the users for the different internal spaces for thermal comfort.

To do so, the system must not only configure membrane patterns based on the different degrees of openness of the ETFE cushions but must also decide when each shading cushion layer will be deployed.

Subsequently, the challenges of artificial intelligence then applicable to the system’s decision-making process design are the following: [21]

(a) Micro worlds. The sum of restricted domains will never be a real environment as follows: the systems should work in a real environment not only in a fully observable domain;
(b) Lack of scalability;
(c) Robustness. Not failing in a novel weather situation is essential, which is why:
(d) Real-time operating is fundamental;
(e) Bottom-up design and embodiment are necessary.

### 4.2. Algorithm Search and Analysis

After the initial considerations, existing intelligent algorithms, from simple to more complex ones, were further examined to check if they were suitable for the implementation of the behavior desired for the façade of the building in our case study.

Francesco Corea’s artificial intelligence technologies classification, the AI knowledge map (AIKM) from 2018, is an outstanding effort to draw an architecture to access knowledge on AI.

The authors developed a diagram of the crossings between the AI paradigms and the AI problem domains. (Figure 6)

The AI paradigms are considered to be the approaches of AI researchers to solve specific problems while the AI Problem Domains are considered to be the problems that AI can historically solve.

Corea points out the following three macro-approaches to AI paradigms:

- Symbolic approach states that human intelligence could be reduced to symbol manipulation;
- Subsymbolic approach is the one in which no specific representations of knowledge are not provided a priori;
- Statistical approach is based on mathematical tools to solve specific sub-problems.

It can be stated then that the architectural discipline will probably deal with sub-symbolic approaches as well as some statistical ones (when working with imaginary perceptions from the environment). As so the search will start from the statistical approach, jumping into the sub-symbolic one. It should be pointed out also that Evolutionary Computation is located within the sub-symbolic only.

As so, the framework for the present research was the only statistical plus sub-symbolic research and optimization. (Figure 7)

As a matter of further research, a framework merely focused on embodied intelligence and hence agent-based modeling and swarm-based optimization are proposed. It is the intention of the authors to compare the results obtained along with the two different frameworks, to be able to draw conclusions from their performance obtained data.

Starting from Corea’s classification and while looking for an algorithm for the system, the parameters established and relevant for deciding the adequacy of an algorithm were initially the following:

- Fully observable vs. partially observable environments adaptability;
- Benign vs. adversarial environments;
- Deterministic vs. stochastic systems;
- Actions of the system being discrete vs. a continuous set of actions, meaning infinite.

Taking all this into account, the search for the algorithm to implement started from the simplest ones to the most complex. In this way, some methods considered were based on probability and statistical theory. (Figure 8)

In this, the following algorithmic processes were discarded as potentially implemented for the façade performance:

*Problem-solving* via *planning:* despite having demonstrated its efficacy within a fully observable environment and within a discrete, deterministic, and known domain, it is not considered a valid method for developing the understanding and decisions related to the learning of weather conditions, as the current study intends to develop the system in a partially observable environment.

*Markov models were discarded* as they are not appropriate algorithms for training memory. Nevertheless, second-order Markov models consider a dependence not only on the previous state but also on the state prior to the previous state. This kind of mathematical model turned out to be rather restricted for the learning the system that is supposed to achieve and was thus ruled out as a possible algorithm.

*Linear Regression and Logistic Regression* use gradient descent algorithms to find local optima. The problem with these algorithms appears when the size of the features array becomes large, because the probability of over-fitting is high, which means having to deal with an extremely large number of parameters. As the system will be dealing with a high number of features, that will make this process clearly very intense and, as such, unachievable.

*Unsupervised learning algorithms,* i.e., *k-means or spectral clustering*, consist of clustering algorithms whose purpose is to find patterns in unlabeled data. [22] Obtaining a large amount of unlabeled data from the environment and trying to find patterns in it to propose new scenarios is a vast improvement in the adaptability behavior of the membrane that might only be considered as a matter of further research when the system has already been proven valid.

*Reinforced learning* via agent analysis is a very effective learning technique. The idea of using the whole system as an agent inside an unknown environment that has to make decisions for a goal and a reward is clearly different from the learning process our system must have, as the concept of the reward function and goal might vary over time during the lifetime of the building. (*see possible further research implementation proposed in the last section).

Machine learning unsupervised algorithms were a good starting point for the configuration of the system, as learning from existing, artificial, or new environmental data models is the main goal of the system, giving the membrane the opportunity to adapt to the environment, learning from it and maximizing its efficacy.

An alternative to logistic regression is to consider a *support vector machine, SVM,* as being a nonprobabilistic linear classifier, which a good alternative to logistic regression. Better error minimization might happen by being trained on the worst classified examples, guaranteeing a smaller error than in conventional logistic regression.

On the other hand, artificial neural networks are likely to work well for most of the settings, but may be slower to train. Accordingly, a priori, it turns out to be the best algorithm to try for the systems’ data management and decision-making from learning from past scenarios.

Considering both algorithms’ implementation for the purpose of testing the abstract digital model of the system and taking into account that a real physical building implementation would not be developed at this stage, the slow training speed was not considered a relevant disadvantage.

### 4.3. Definition of the Artificial Neural Network and Evolutionary Computation Model

ANNs are normally adaptive to the external environment [23] as they develop their learning from the data received from it. Modern neural networks are non-linear statistical data modeling tools [24] trying to simulate the brain’s parallelism way of working and learning capability by training and pattern recognition by feed-forward and back propagation. Therefore, loop networks with feedback and the idea of backpropagation are the definitive alternatives for adaptability. This kind of machine learning algorithm gives the system the ability to distinguish between different kinds of environments and situations, past performances, and storage labeled data.

The process is proposed as a dual one and will need the power of neural processes for choosing and decision-making situations, but also the performance of a genetic algorithm for optimizing the cushion pattern of the membrane and its adaptability.

Firstly, an initial process that could be able to recognize through the ANN a thermal situation similar enough to the one in the database of past performances.

Secondly, if no labeled alternatives are found, a new dynamic process for thermal adaptation to the new scenario will be performed. Every scenario is always composed of users’ thermal requirements, external temperature measurements, and shading situations for every cushion. The combination of both sub-processes will generate global behavior in which, from the very first instance after deployment, the system will perform properly.

The system’s algorithm will be composed, as shown in Figure 9, as follows:

An ANN for classifying and deciding the kind of scenario the system is in (learned through a series of labeled sets of situations for training)

A genetic algorithm that will optimize the performance of the whole set of the ETFE cushions, creating a pattern for adaptability and improvement.

Phenotype, genotype, fitness, and mutation will decide and teach the system how to act in each situation (global behavior) but will also propose when to deploy the layer for solar gain control (Local cushion behavior).

### 4.4. Artificial Neural Network Definition

The first decision for the learning behavior enables the membrane to decide in which scenario it is working in accordance with pre-trained scenarios. Then, the performance customization of the insulation layer is performed on-site through the internal environmental control procedure implemented.

The proposed ANN will have 100 neurons in the input layer and 150 neurons in the hidden layer. Each one of those 100 neurons corresponds to the behavior and temperature of each cushion of the membrane. The proposed outputs will be a set of different performances of the membrane, optimized for the previous run of the genetic algorithm (GA). Thus, depending on the output, one kind of behavior for insulation or another will start. Then, in case some pattern of opening and closing cushions is found to be more efficient than the current one, the membrane will be readjusted (Figure 10). Once a minimum amount of training has been performed, we can test the learning of our system.

The training set will be a total of 10.000 arrays of 100 elements, each of which will be a possible temperature in the scenario the array is labeled (for this particular research, most of the temperature data used in the training came from the International Research Institute for Climate & Society, the IRI Climate Data Library (*IRI-LDEO Data Library*) as no previous learning patterns exist.) After defining how to load the data from the *.dat files previously created with the encoded byte info, the inputs and outputs arrays will be defined, as well as the behavior of one neuron. The input temperatures will be scaled for working within the network as floating points between -1 and 1. In another class, the network behavior will be described, ordering the steps for the back propagation and always checking the errors of the previous layer). The complete training set will be an array stored as a *.dat file of [10,0].

Training set = 10,000 pseudo set

New pseudo set = new float [100]; for ex labelled as scenario 1.

Pseudo set[i] = possible temperature in the scenario 1.

Every time the network is running from the beginning of the process, a new training set is created. The network will train when started, and it will initially read a set of 500 training labeled arrays. For extra training afterward, a function will be implemented that when pressing a key, another 10 training sequences take place, as shown in Figure 11 below.

The simplest output implementing the unit step function, which is a function whose value is 1 for negative arguments, and 0 for positive arguments, is considered too simple. As such, a differentiable function implementation is necessary. The activation function of the nodes will thus be the following:

Output = 2/(1 + e^−2^*^inputs^) − 1 (2)

The testing time decided on for checking the input temperature pattern will be every 30 min, a period during which the ANN will re-decide within which scenario it is located and will re-apply the genetic algorithm, if necessary.

### 4.5. Configuration of the Abstract Model and Interface

The creation of the abstract sample of the facade requires an *object-oriented programming, OOP,* language. The chosen language will be Java through *Processing* (a flexible Java-based) The aim is also to be able to create a graphical interface easily understandable on the screen for the purpose of appreciating the behavior of the facade along the implemented process.

It will be designed with a very simple layout/interface of the performance so it can appreciate, in an easy and straightforward way, the behavior of the cushions in every test.

As such, the abstraction of the facade will be composed of one hundred ellipses that will change color on the model, depending on its degree of openness. As shown in the figure below, grey will represent the ETFE cushion when completely open, and white when the cushion is completely closed. The intermediate sizes of the ellipses will represent the different degrees of opening.

The intermediate shading layer that every cushion has will be represented by a set of small grey ellipses within each cushion and will only appear when the layer for shading is open (any time the solar factor detected is higher than FS 10.) (Figure 12)

Moreover, the final temperature obtained as a result of the process in the space right behind that pillow is written down on them for better visualization of the results.

### 4.6. Facade’s Chromosome and Genes Array Definition

For the purpose of the creation of the phenotype of the abstract model of the facade, a total of 100 ETFE cushions configuring the chromosome will be considered.

As so, a chromosome of the facade through an array is proposed, Genes [], which is a sequence of all opening and closing possibilities for the 100 ETFE cushions that configure the facade abstract sample that is studied.

The genotype is thus an array of 100 elements that indicates the initial position of the cushions the system is starting with.

The positions considered initially for the facade cushions are the three following: closed (c), open (o), and half-open (h).

Genes = new [100]; Genes [i]= [open/close status](3)

Membrane genotype = [c, o, c, h, c, c, h, o, c...](4)

This “species” of the facade will have to evolve and adapt to different users’ requirements and weather conditions through the adaptation of its phenotype expression as follows: the façade ETFE cushion working patterns.

An initial test was set up for optimization of the genes’ array of the facade to obtain a desired homogeneous temperature of 22° in all internal spaces behind the abstract sample of the facade. Possibilities for the genes were constrained to the three options mentioned until the system has been tested to work properly.

### 4.7. Phenotype Definition and Fitness Function Implementation

For the system, the environment in which this phenotype exists is the thermal relationship between the environment and the material.

The phenotype will vary depending on the different behavioral patterns the facade will perform according to the different thermal requirements.

Several simple fitness functions will be implemented, based on the idea of obtaining an ideal temperature for each cushion of the facade.

The trials were performed with a percentage of mutation of between 0.01 and 0.05%, meaning that then the probability of mutation is as follows:

mutation probability = 1/chromosome length [0.01–0.05%](5)

Then, the thermal relationship between the degree of openness of the cushions and the temperature variability was implemented as the fitness function in the algorithm.

After implementing the genotype-phenotype relation, a fitness function included trying to prevent premature convergence and stagnation. This function is also related to thermal behavior and will be implemented in the phenotype for each of the 100 cushions as follows:

ΔTemp = (Q × thickness)/λ(6)

t_f [i] − temps[i] = G × genes[i]/λ(7)

Being the following:

t_f [ ] final temperatures array.

temps[ ] input temperatures array at the beginning of the optimization process.

### 4.8. Selection Method and Pareto

The method implemented initially was Alasdair Turner’s [25] interpretation of the rank selection. Starting with a fitness smaller than −2000, stagnation appears with a fitness of −727 around evolution number 1000.

A study of the implementation of different selection methods was carried out to improve the genetic algorithm performance. An improvement to the maximum fitness was obtained, −727, by using different combinations of selection methods and variations of the current fitness function.

The roulette wheel selection will not be implemented due to the danger of premature convergence it generates if a clearly dominant individual exists, which could be the case. The methods considered are top scaling and, as a second option, tournament selection.

The implementation of the tournament selection method improves the performance of the algorithm. Nevertheless, the experiment only proved to increase fitness by 0.98%. When the top scaling selection was implemented, the fitness decreased by 1%. Tournament selection will, therefore, be the method used (Figure 13).

As the fitness function should improve after choosing the most effective selection method, the Pareto frontier will be implemented.

Considering the optimization of two values, *Q*, the t*hermal flux*, and *t_f*, the final temperature, as follows:

Objective A → Thermal flux min

Objective B → t_f = 22 °C

Weights proposed W_A_ → 0.2 and W_B_ → 0.8

Fitness function → f(x)= 1/(1 + Wa × A + Wb × B)(8)

A homogeneous, controlled decrease in temperatures has taken place across the whole membrane as desired in the inputs of the program. However, because of the thermal property relationship implemented, (7), and the constraints on the degree of openness of the cushions (8), the results obtained in this experiment showed a maximum fitness of 0.016, optimizing the maximum insulation properties of the material and of the ETFE cushions (Figure 14).

T_f [i]= Q × genes[i]/0.017 + Temp [i](9)

genes [i] = 0 genes[i] = 0.5 or genes [i] = 1(10)

## 5. Results

After implementing the thermal relationship of the material in the façade performance, a complete process loop is performed, and, as so the constraints of the degree of opening for the cushions are removed.

The different membrane patterns created by the learning of the system as well as the local behavior for the shading layer of the cushions, successfully optimized the material insulation performance of the system, thus maximizing material insulation properties.

When the final non-constrained openness of the cushions is allowed, the maximum efficacy of the insulation properties of the designed façade is reached. Previously, implementing the rank selection method stagnates fitness in a −727 situation that improved by implementing tournament selection, obtaining a final fitness for the system of 0.016 after 24 h of training and 10,000 iterations.

When considering a free degree of opening possibilities for the cushions, from thickness 0 to thickness 1, in which initialization will be the following:

genes [i] = random (0, 1)(11)

* being all floats in [0, 1] allowed as possible degrees of openness maximum optimization of the material will be achieved.

**Figure 15:**
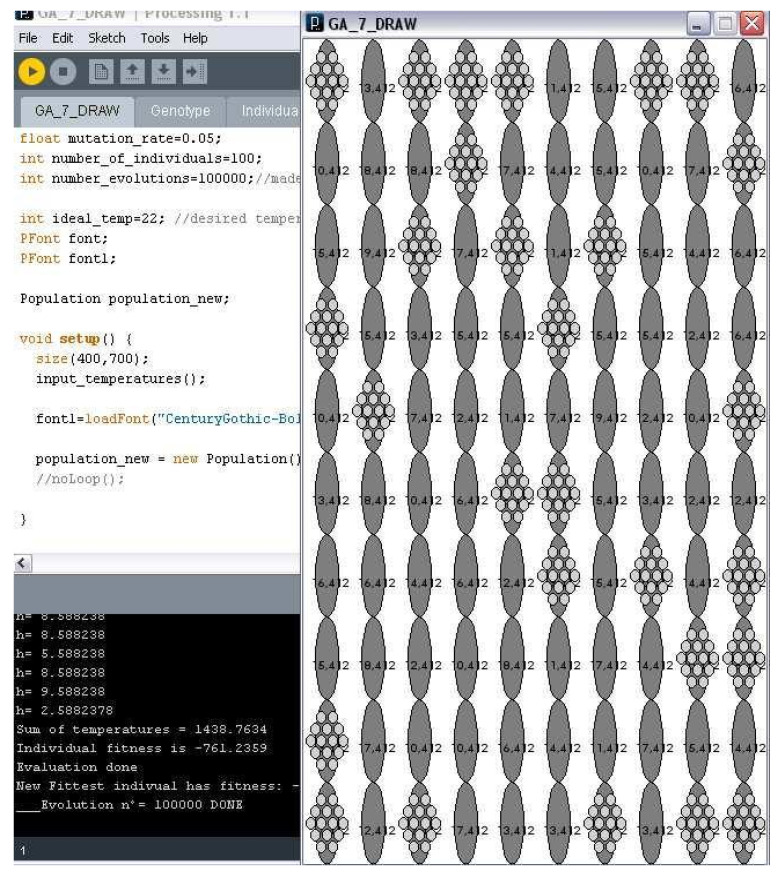
Results shown by no degree of openness restrictions.

As so, in order to prove that maximum fitness is reachable, a different code with a nonconstrained gene array of the opening was run. After a certain time of working, the code reached the maximum fitness, a fitness of 1, demonstrating that the possibility of optimizing the insulation of the ETFE is real. (Figure 15). The proposed process did prove viable, efficient, and scalable.

The results are viable for comparison when any other method that may be introduced in the future. As so, the statistical evaluation will be able to be performed along with the results of the other models to be implemented in the future, as mentioned in the further research section.

## 6. Conclusions

Although the set of different layers proposed for the facade’s configuration has been demonstrated to be insufficient for proper insulation and climate regulation, results and further tests proved the system tested for its analysis is a valid and efficient process.

Therefore, it can be concluded that the settings, phenotype model, and workflow proposed by the authors have the capability and potential to succeed in all the scenarios required. The combination of the methodology and the evolutionary computation’s algorithm executed provides new possibilities to address real-time decision-making for dynamic thermal insulation in similar facade systems.

Likewise, embedding dynamic systems through novel AI algorithms-based abstract models has been proven relevant. Moreover, unlike other algorithms and because of its exposure to different circumstances and environments, it will get better and faster as time goes by, improving all along with the building’s lifespan.

As the authors proposed at the beginning of the text, “time” can be stated then as the parameter that intrinsically links aesthetics with lifetime performance.

This is fundamental for an architecture that is self-aware, adaptable, responsive, and agile, where real-time aesthetics are directly related to operations

The system’s final form is dynamic and will be changing depending on user-environment requirements, linking directly through “time” user-system-aesthetics efficacy and insulation optimization.

## 7. Further Research: Distributed Artificial Intelligence Implementation

- *Strict geometrical calculation of gas volume in the ETFE’s cushions*

One of the first improvements to be addressed in the future is the strict calculation of thermal performance by proper environmental software. This calculation should be carried out by a specialized engineer. Flat surfaces, as in this work for simplification, should not be assumed but the different curvature degrees of the cushions in each phase of the opening-closing procedures should be considered.

For example, when considering a thin material, differential r, and a radius r, supposing a cylindrical curvature of the cushions, the thermal resistance considered should be, approximately the following:

R = 1/2 L × Ln (r_2_/r_1_) for r_2_ > r_1_ (12)

- *Variable needs requests from internal users, rooms, and weather scenarios*

Moreover, fundamental further research must be developed regarding the connection between the inner environmental variables (heat gain, loss, lumens…) and different façade patterns. The current research presents just a regularly designed pattern applied to a unique inner space. Moreover, further research is needed, based on training the whole software for all the weather scenarios possible, introducing all possible variabilities for the implemented Genetic Algorithms.

As so, the dynamic environmental system proposed can complete its design by improving training paths and testing different weather scenarios.

Dynamism in real-time and inaccessibility to environmental data could be one of the limitations of the system if this particular aspect of the further research proposed is not developed.

- *Distributed AI. Embodied Intelligence implementation.*

As it was stated in the methodology chapter, the research will be implemented through methodologies of distributed intelligent systems as the second methodological analysis, which result will be used for the final comparison and results.

As an alternative, a Multiagent Self-Organized Agent System will also be adequate for the testing of the full weather scenario. A potential basis could be to consider the facade’s system as the performance of a set of autonomous agents that improve its behavior and adaptability in reaching their goal through practice and experience [26].

i.e., a Q-Learning algorithm seems to be an appropriate approach for future developments. These agents, each of which is considered as a cushion of the facade, can shape their behavior through learning in accordance with the environmental context, including a reward function.

The proposed method would work in a diagram such as the one proposed by Calderoni and Mancenac [27] at the 12th Artificial Intelligent Conference in their paper “*MUTANT: A genetic learning system*”. Their paper, presented in 1999, demonstrated the efficacy of the application of Q-Learning in a situation such as the one presented in this paper. Agent simulation and encoded GA as the basis of the rules of agents’ behavior are proposed as the initial ideas for further development of the intelligent system.

As reinforced learning agents then, the ETFE cushions can explore the environment through sensors and light meters. They can find where and what the rewards are and know the best policy to move towards them. At the initial stage of learning, the agents always return to the initial fixed values decided by the designer. The environment is therefore unknown at the beginning of the learning, which is exactly the kind of situation the membrane will be in on arrival at the site.

It is considered of special value to make future comparisons between the three algorithms mentioned (ANNs vs. Agents vs. Q-Learning), considering their effectiveness against the adaptability to weather conditions.

## Figures and Tables

**Figure 5:**
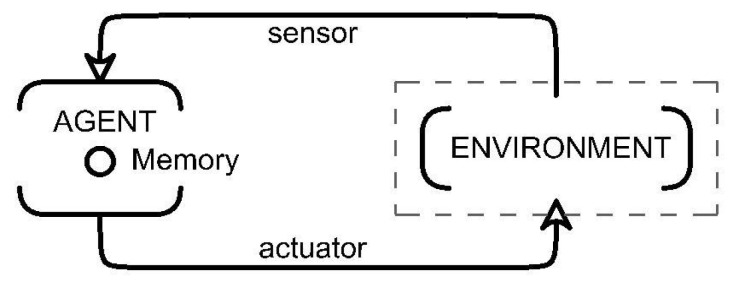
Intelligent agent loop proposed. Source authors.

**Figure 6:**
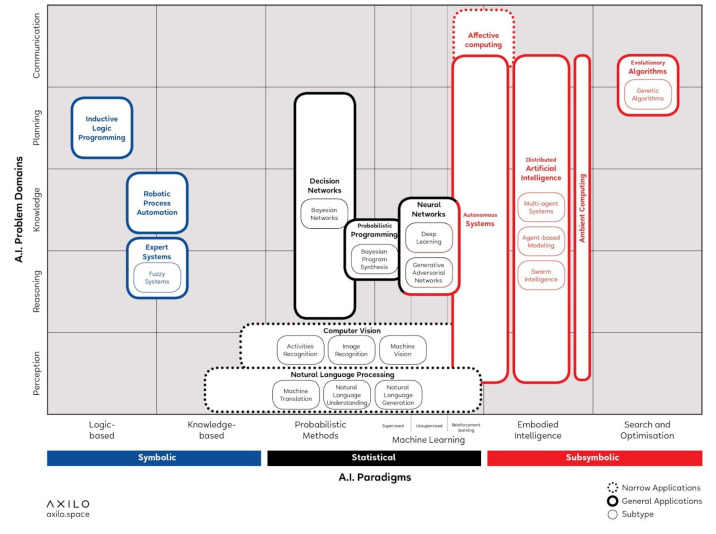
AI knowledge map (AIKM developed by F. Corea for the strategic innovation consultancy Axilo, for activities on their Chôra platform.).

**Figure 7:**
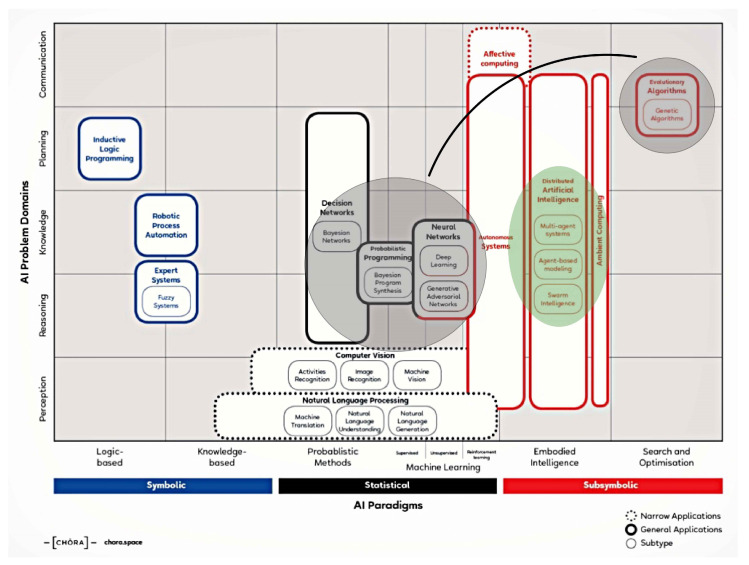
Framework of research. Present research, statistical + subsymbolic framework(grey); further research (green) embodied intelligence framework.

**Figure 8:**
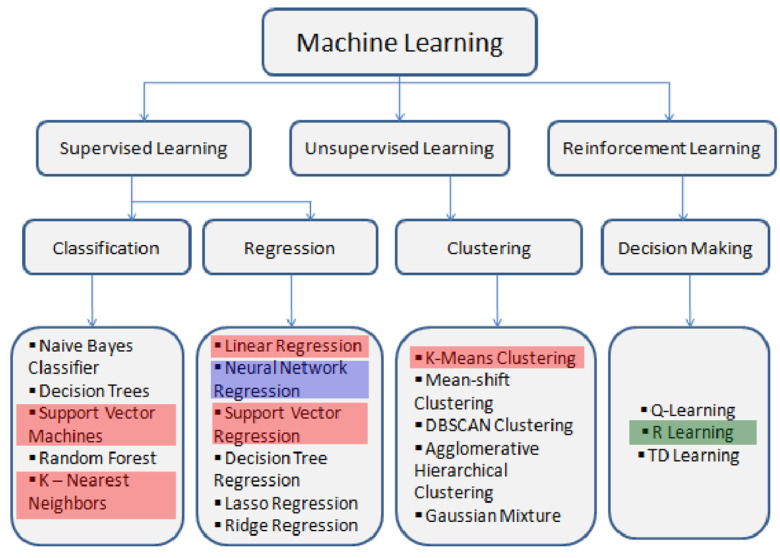
Analyzed algorithms (red); further research implementation (green); implemented along this paper (blue).

**Figure 9:**
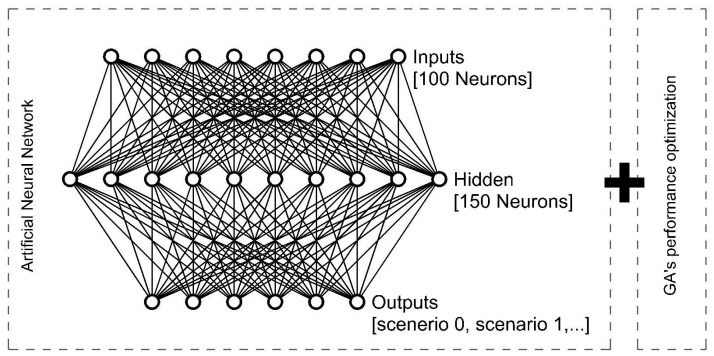
Final artificial neural network structure proposed.

**Figure 10:**
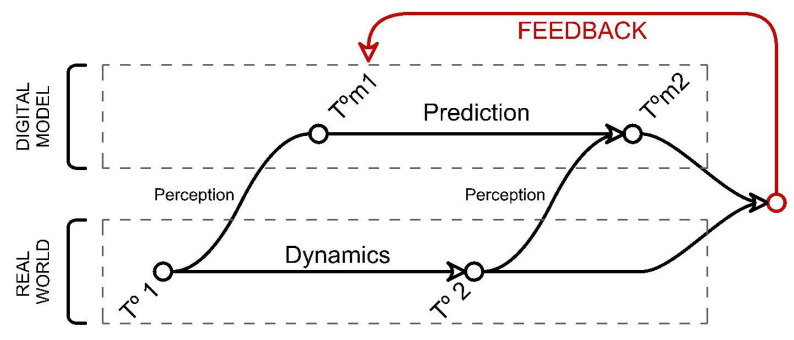
Learning cycle proposed.

**Figure 11:**
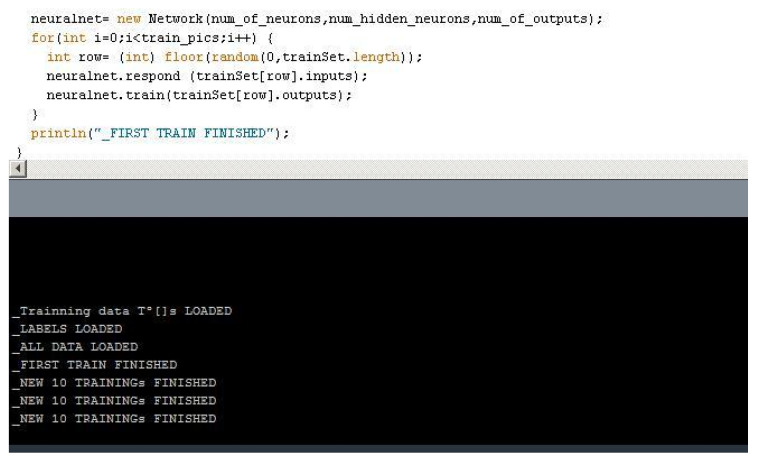
Training process sample code.

**Figure 12:**
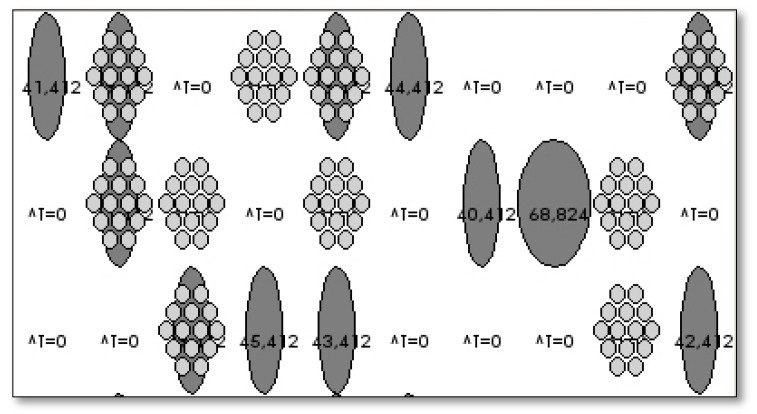
Typical representation of the facade on the abstract model/interface.

**Figure 13:**
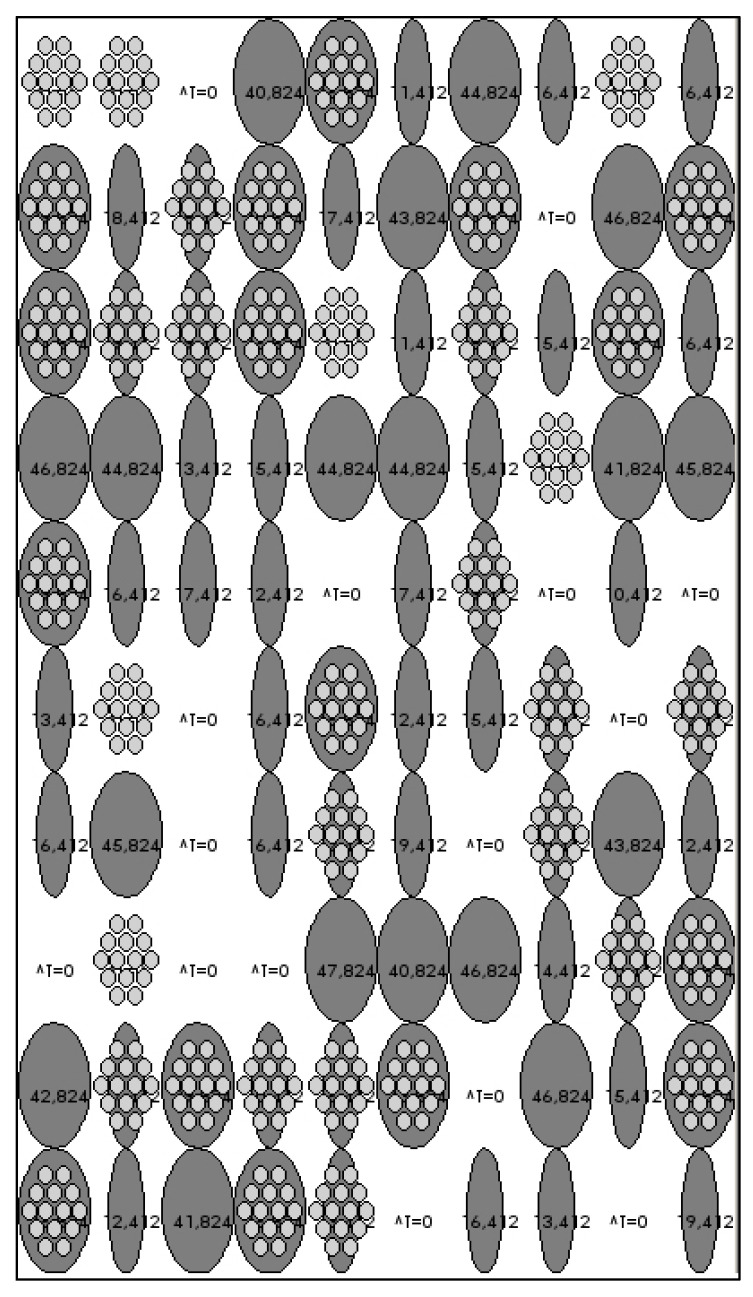
Initial tests results with tournament selection implementation.

**Figure 14:**
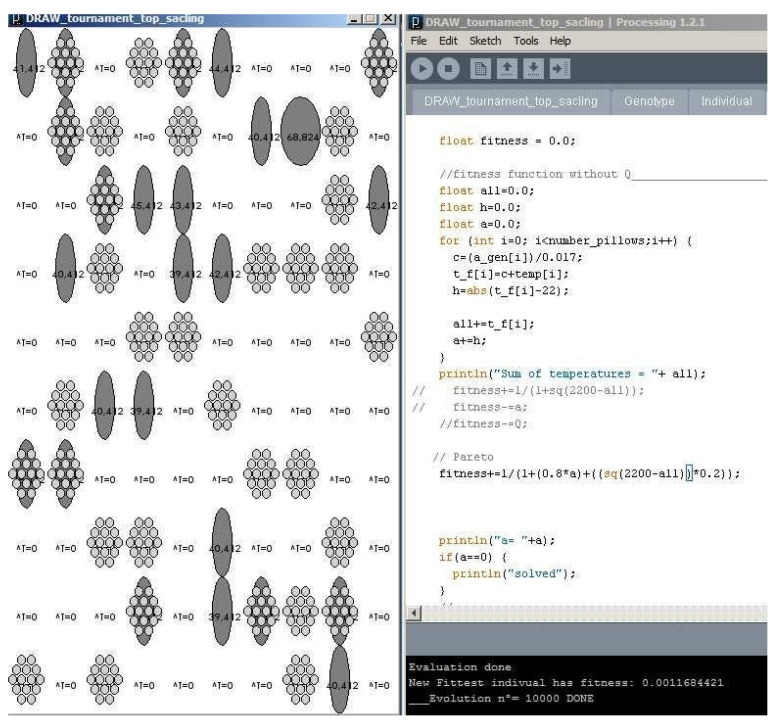
Results for the 10,000th generation of the membrane performance.

## Data Availability

Not applicable.
